# Scan for Success? Number Needed to Scan and Management Changes in Preoperative Otosclerosis: Quantitative Analysis

**DOI:** 10.1002/ohn.70320

**Published:** 2026-06-23

**Authors:** Omer J. Ungar, Adi Brenner, Daniel Zikk, Rani Abu‐Eta, Yahav Oron, Oren Cavel, Ophir Handzel, Nidal Muhanna, Shiri L. Maymon

**Affiliations:** ^1^ Departments of Otolaryngology–Head and Neck Surgery and Maxillofacial Surgery, Tel Aviv Sourasky University Medical Center, Gray Faculty of Medicine Tel‐Aviv University Tel Aviv Israel; ^2^ Departments of Radiology, Tel Aviv Sourasky University Medical Center, Gray Faculty of Medicine Tel‐Aviv University Tel Aviv Israel; ^3^ Hôpital universitaire des enfants reine Fabiola, H.UB, université libre de Bruxelles Bruxelles Belgium; ^4^ Department of Otolaryngology University of Rochester Rochester New York USA

**Keywords:** enlarged vestibular aqueduct. persistent stapedial artery, lateral ossicular chain fixation, otosclerosis, overhanging facial nerve, superior semicircular canal dehiscence

## Abstract

**Objective:**

To quantify the cumulative number of images needed to scan (NNS) with preoperative high‐resolution temporal bone computed tomography (HR‐TBCT) to yield a change in management in patients undergoing primary stapes surgery for otosclerosis‐related hearing loss.

**Study Design:**

Retrospective cohort.

**Setting:**

Tertiary academic referral center.

**Methods:**

Medical records of all patients referred for primary stapes surgery between 2010 and 2025 were reviewed. All patients suspected of otosclerosis routinely underwent preoperative HR‐TBCT scan, and their CT findings were compared to the intraoperative findings of those who underwent surgery. All findings that altered informed consent, surgical candidacy, or surgical approach were recorded. The cumulative and temporal bone‐specific condition NNS were calculated.

**Results:**

A total of 892 patients were identified (male‐to‐female ratio 305:587). HR‐TBCT revealed temporal bone pathology resulting in management change in 14% (1^24^ ears). Prevalence of temporal bone conditions that alter management of stapes surgery included third window lesions (5.8%), lateral ossicular chain fixation (4.1%), overhanging facial nerve (1.3%), obliterative otosclerosis (1.0%), far‐advanced otosclerosis (1.0%), and persistent stapedial artery (0.6%). The cumulative NNS for at least one management change, confirmed intraoperatively, was 38 (95% confidence interval 77‐22).

**Conclusion:**

Routine preoperative HR‐TBCT identifies clinically significant temporal bone pathology that alters management in approximately 14% of stapes surgery candidates. The NNS for intraoperatively confirmed findings was 38. HR‐TBCT facilitates more accurate patient selection, improves informed consent, and reduces intraoperative complications. Incorporating HR‐TBCT into the preoperative assessment enables comprehensive presurgical counseling and planning.

Stapes surgery is a well‐established treatment option for patients with otosclerosis who seek hearing improvement but prefer not to use a hearing aid. While the definitive diagnosis of otosclerosis is confirmed intraoperatively, clinical suspicion supported by audiological findings typically provides sufficient justification for middle ear exploration.

Several temporal bone entities have been associated with poor surgical outcomes, intraoperative abortion, or prolonged operative time, including superior semicircular canal dehiscence (SSCD), an enlarged vestibular aqueduct (EVA), a persistent stapedial artery (PSA), lateral ossicular chain fixation, and an overhanging facial nerve (FN).^1^, ^2^, ^3^ Several of these conditions can be identified on preoperative high‐resolution temporal bone computed tomography (HR‐TBCT). Despite this, imaging has not traditionally been considered essential for the management of otosclerosis‐related hearing loss, even though it may provide valuable information in the preoperative evaluation of candidates for stapes surgery. The precise value of CT scans in the setting of otosclerosis is still a matter of controversy in clinical practice, and patients with a typical history do not routinely undergo systematic radiological assessment prior to surgery.^4^ Importantly, specific CT findings may influence the surgical plan and alter the informed consent process.

The objective of this study is to quantify the cumulative number needed to scan (NNS) for a single management change of at least one of the following: informed consent, surgical candidacy, or surgical approach in patients undergoing primary stapes surgery for otosclerosis‐related hearing loss. This study provides evidence‐based estimates of the diagnostic and clinical yield of routine imaging in this setting, and for the risk of not scanning. Hopefully, this data will reduce the number of aborted surgeries and the intraoperative complication rates as a result of intraoperative findings that could have been detected preoperatively by means of HR‐TBCT.

## Methods

### Study Design and Ethical Considerations

This was a retrospective cohort study conducted at a single tertiary referral center. The Sourasky Medical Center research ethics committee approved the study protocol (0563‐23‐TLV) and waived the requirement for informed consent since all the data were anonymized.

### Setting and Patients

The study included all consecutive adult patients (≥18 years‐of‐age) diagnosed as having or treated for otosclerosis‐related hearing loss between 2010 and 2025. Eligible cases were identified through International Classification of Diseases, Ninth‐Revision codes.

### Imaging Protocol

HR‐TBCT is mandatory prior to middle ear exploration in our institution. The indication for HR‐TBCT is not for the diagnostic confirmation of otosclerosis, but rather for preoperative assessment of contraindications, surgical planning, and informed consent discussions (Figure 1). CT examinations were performed using various multidetector CT scanners (32‐256 slice systems), with slice thickness between 0.4 and 0.6 mm. Images were acquired in the axial plane, with multiplanar (three‐plane) reconstructions generated for optimal evaluation. Each axial CT‐scan was reconstructed in the coronal and sagittal planes, as well as in Stenvers and Pöschl planes, if needed, utilizing the Advantage Workstation (GE‐Healthcare). The selection of the planes was based upon the plane bisecting the internal acoustic canal and the lateral semicircular canal. All CT‐scans included the bone window images that were used for this study. All scans were reviewed by both a neuroradiologist and otologist/neurotologist.

**Figure 1:**
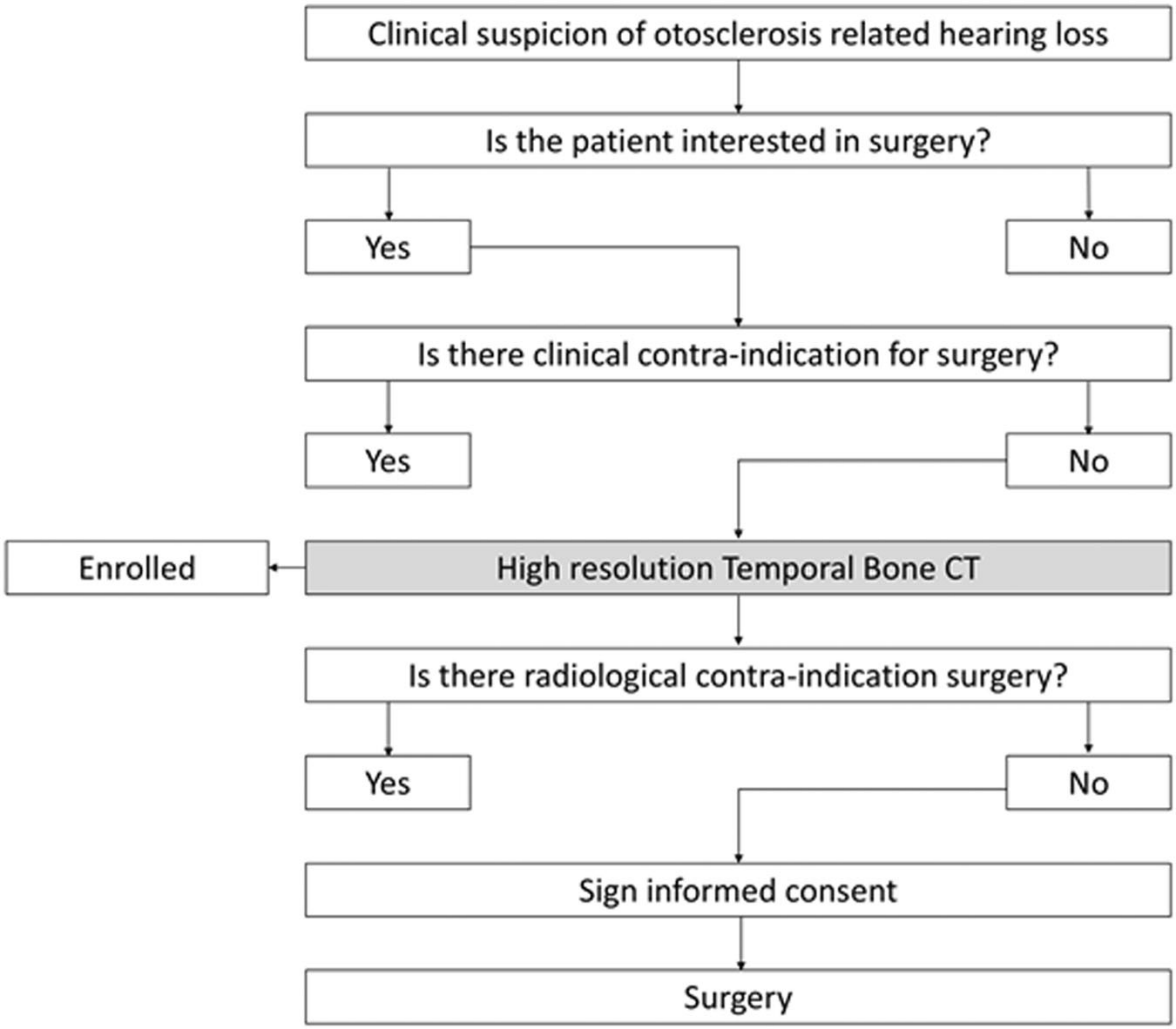
Preoperative assessment protocol of a surgical candidate with otosclerosis‐induced hearing loss. High‐resolution temporal bone CT scan is mandatory according to the protocol. CT, computed tomography.

### Inclusion and Exclusion Criteria

Patients were included if they elected surgical treatment for otosclerosis and had an available HR‐TBCT scan in their electronic medical record. They were excluded if they declined surgical intervention or were lost to follow‐up before undergoing HR‐TBCT.

### Data Collection

Data were extracted from electronic medical charts, and they included the findings from HR‐TBCT scans and surgical reports. Some radiological findings, such as EVA and SSCD, can**not** be confirmed intraoperatively. Moreover, some patients whose TB conditions suggested non straightforward stapes surgery had declined surgery and their TB conditions could not be verified.

The intraoperative findings were used as the gold standard (reference), reasoning that it is the best available method for determining the true disease status. These findings were compared to those of the HR‐TBCTs, which was defined as the tested diagnostic modality. These parameters were used to calculate all diagnostic accuracy measures: specifically, sensitivity, specificity, false positive and negative rates, positive and negative predictive values, false omission and discovery rates, prevalence, likelihood odds ratio and NNS.

### Statistical Analysis

Categorical variables were summarized as frequencies and percentages. Age distribution was assessed for normality using histograms. Since age was approximately normally distributed, it was reported as the mean ± standard deviation (SD). The mean age was compared between males and females using an independent‐samples *t*‐test. Sensitivity and specificity were calculated, and the 95% confidence interval was evaluated using the simple *Z* with continuity correction method. The NNS was calculated as 1/(Sensitivity × Prevalence). All statistical tests were 2‐tailed, and statistical significance was defined as a *P* < .05. The data were compiled in Microsoft Excel and analyzed using NCSS software (NCSS 2025 Statistical Software, NCSS, LLC.).

## Results

The medical charts of 892 candidates for stapes surgery with the clinical diagnosis of otosclerosis‐related hearing loss were enrolled. The male‐to‐female ratio was 305:587 (~1:2). The average (±SD) age was 49.3 (±11.6) years and did not differ significantly between sexes (49.0 and 50.0 for females and males, respectively, *P* = .245).

Upon their being considered candidates for surgery, all of the study patients underwent HR‐TBCT as a part of the preoperative assessment, and 724 (86.2%) of the imaging study results supported straightforward stapes surgery. In addition, 124 (13.8%) of the imaging studies detected a temporal bone (TB) condition that could modify the surgical approach, postoperative outcome, surgical candidacy or informed consent (the “group of interest”). 37(29.8%) of the latter group of patients were diagnosed as having nonotosclerosis fixation of the ossicular chain (most commonly fixation of the head of the malleus), followed by SSCD (*n* = 34, 27.4%) and EVA (*n* = 18, 14.5%). Combined, third window lesions (ie, SSCD and EVA) were diagnosed in 52 (41.9%) of the patients in the group of interest. An overhanging FN was revealed in 12 (9.7%) of the group of interest, followed by far advanced (*n* = 9, 7.3%) and obliterative otosclerosis (*n* = 9, 7.3%). PSA was diagnosed in 5 (4.0%) of these cases (0.6% of the entire cohort). The incidence of the various TB conditions in the entire cohort and in the group of interest is provided in Table 1 and Figure 2.

**Table 1:** Incidence of CT Findings Suggestive of Complicated Intraoperative Course and/or Poor Outcome

	Entire cohort	Group of interest
Preoperative CT was not beneficial (%)	86.2	NA
Preoperative CT was beneficial^a^ (%)	13.9	100.0
Lateral ossicular chain fixation (%)	4.1	29.8
Superior semicircular canal dehiscence (%)	3.8	27.4
Enlarged vestibular aqueduct (%)	2.0	14.5
Overhanging facial nerve (%)	1.3	9.7
Obliterative otosclerosis (%)	1.0	7.3
Far advanced otosclerosis (%)	1.0	7.3
Persistent stapedial artery (%)	0.6	4.0
Total (%)	100.0	NA

Abbreviation: CT, computerized tomography.

^a^ Changed surgical candidacy, surgical plan, or informed consent.

**Figure 2:**
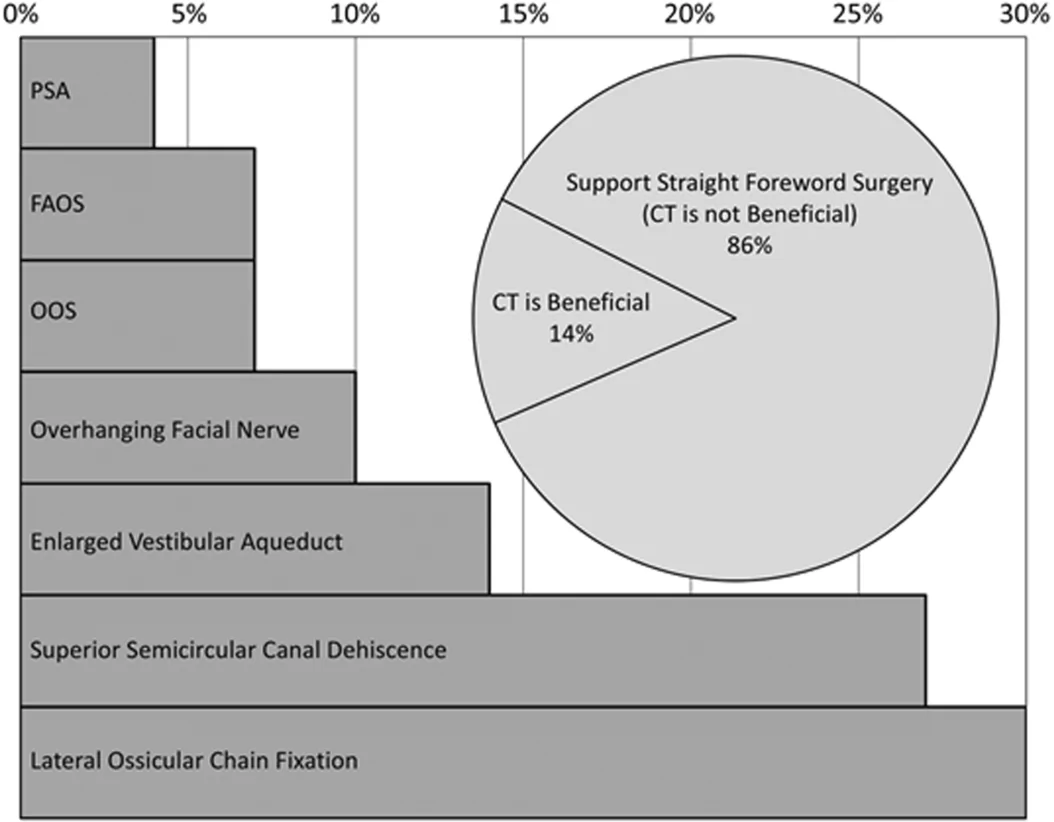
Likelihood of beneficial versus nonbeneficial preoperative CT scan (pie chart). Substratification of beneficial CT scan is shown as a histogram. CT, computerized tomography.

In order to study the overall cumulative sensitivity and specificity of HR‐TBCT for any TB condition, we compared the radiological diagnosis to the intraoperative findings. Overall cumulative sensitivity and specificity of HR‐TBCT was 451 and 904, respectively (95% confidence interval [CI]: 305‐0.597 and 904‐0.883, respectively). All other parameters for the various TB conditions as well as the cumulative level of diagnostic CT accuracy are listed in Table 2. The sensitivity and specificity of HR‐TBCT for lateral ossicular chain fixation was 500 and 966, respectively (95% CI: 224‐0.776 and 953‐0.979, respectively). The sensitivity and specificity of HR‐TBCT for an overhanging FN was 375 and 998, respectively (95% CI: 107‐0.644 and 994‐>0.999, respectively). The sensitivity and specificity of HR‐TBCT for PSA were 1.0 for both (95% CI: 833‐>0.999 and >.999‐>.999, respectively). The sensitivity and specificity of HR‐TBCT for obliterative otosclerosis was 375 and 997, respectively (95% CI: 107‐0.644 and 992‐>0.999, respectively). The NNS could be calculated only for the participants who underwent surgery. Since a third window is considered a contraindication for stapes surgery in our institution, and given that far‐advanced otosclerosis cannot be confirmed intraoperatively, the extent of diagnostic CT accuracy cannot be extrapolated for these entities.

**Table 2:** Diagnostic Accuracy Measures of CT Scan for the Analyzed Temporal Bone Conditions

	Overall			Lateral ossicular chain fixation			Overhanging facial nerve			Persistent stapedial artery			Obliterative otosclerosis		
		95% CI limit			95% CI limit			95% CI limit			95% CI limit			95% CI limit	
Measure	Value	Lower	Upper	Value	Lower	Upper	Value	Lower	Upper	Value	Lower	Upper	Value	Lower	Upper
Sensitivity (TPR)	0.451	0.305	0.597	0.500	0.224	0.776	0.375	0.107	0.644	>0.999	0.833	>0.999	0.375	0.107	0.644
Specificity (TNR)	0.904	0.883	0.925	0.966	0.953	0.979	0.998	0.994	>0.999	>0.999	0.999	>0.999	0.997	0.992	>0.999
Sensitivity + specificity	1.355	NA	NA	1.466	NA	NA	1.373	NA	NA	2.000	NA	NA	1.372	NA	NA
False negative rate	0.549	0.403	0.695	0.500	0.224	0.776	0.625	0.357	0.894	0.000	<0.001	0.167	0.625	0.357	0.894
False positive rate	0.096	0.076	0.117	0.034	0.021	0.047	0.002	0.000	0.006	0.000	<0.001	0.001	0.004	<0.001	0.008
Positive predictive value	0.226	0.140	0.312	0.216	0.070	0.362	0.750	0.387	>0.999	>0.999	0.833	>0.999	0.667	0.303	>0.999
Negative predictive value	0.964	0.950	0.978	0.990	0.983	0.998	0.988	0.981	0.996	>0.999	0.999	>0.999	0.988	0.981	0.996
False omission rate	0.036	0.023	0.050	0.010	0.002	0.017	0.012	0.004	0.019	0.000	<0.001	0.001	0.012	0.004	0.019
False discovery rate	0.775	0.689	0.861	0.784	0.638	0.930	0.250	<0.001	0.613	0.000	<0.001	0.167	0.333	<0.001	0.697
Prevalence	0.059	0.042	0.075	0.018	0.009	0.028	0.018	0.009	0.028	0.003	<0.001	0.008	0.018	0.009	0.028
Likelihood odds ratio	7.705	4.236	14.016	28.483	9.991	81.199	255.900	45.940	1425.436	0.000	<0.001	<0.001	170.400	37.281	778.847
Number needed to scan	**38**	77	22	**109**	502	46	**145**	1055	56	**294**	1200	127	**145**	1055	56

Abbreviations: CI, confidence interval; TNR, true negative rate; TPR, true positive rate.

The overall cumulative NNS (95% CI), which represents the number of patients needed to undergo a CT‐scan prior to stapes surgery in order to indicate the need for a management change in a single patient was 38 (77‐22). The NNS (95% CI) of the various TB‐specific CT findings was found to be 109 (502‐46) for lateral ossicular chain fixation, followed by 145 (1055‐56) for an overhanging FN and obliterative otosclerosis, and 294 (1200‐127) for PSA (Table 2).

## Discussion

While clinical suspicion of otosclerosis is based upon history taking and physical examination, confirmation is routinely performed intraoperatively. The introduction of HR‐TBCT more than 3 decades ago abruptly replaced the conventional pluridirectional tomography^5^ and became an integral part of the assessment of various TB conditions.

While preoperative HR‐TBCT in the context of cholesteatoma excision^6^ or cochlear implantation^7^ is widely accepted, the role of HR‐TBCT prior to middle ear exploration for clinically suspected otosclerosis is less clear and therefore not widely accepted. In fact, a recent survey reported that it was used routinely by fewer than 50% of surgeons who are active members of the American Neurotologic Society and American Otologic Society.^8^ The sensitivity of CT for the diagnosis of otosclerosis is highly dependent upon the diagnostic modality that is used as a reference. For example, the sensitivity of CT for the diagnosis of otosclerosis compared to the intraoperative finding of otosclerotic stapes fixation is generally in the range of 75% to 95%,^9^, ^10^, ^11^, ^12^ dropping to 60% to 65%^13^, ^14^ when compared to histopathological preparations. Some authors believe that the routine use of CT scans in otosclerosis is still not well‐established due to the rarity of concomitant malformations of the inner ear, the low predictive value of CT for intraoperative gushers, the absence of surgical contraindication in extensive multifocal disease, the associated costs, and the inherent exposure to radiation.^4^, ^15^

One of the reasons for these beliefs may derive from confusion concerning the indication for an HR‐TBCT scan. Many studies had focused upon the diagnostic yield of preoperative HR‐TBCT, some of which were compared and analyzed by Wenger et al., 2016.^16^ After pooling the data, those authors concluded that “Pre‐operative CT has little to add in establishing otosclerosis and may not be necessary to confirm the diagnosis.” This statement is currently widely held. However, the indication of HR‐TBCT is different when a patient with clinically suspected otosclerosis‐related hearing loss is being evaluated for surgical candidacy. The questions then center around candidacy for undergoing the procedure, surgical planning, and informed consent adjustment.

In 2018, Wolfovitz and Luntz^17^ provided a checklist for the assessment of preoperative HR‐TBCT. The cost of TBCT imaging varied considerably between regions. In Europe, CT‐scans are generally inexpensive, whereas the average cost is approximately $1450 in the United States.^18^ These financial considerations need to be factored into the decision‐making process regarding preoperative imaging. The findings of our study define the required NNS in the specific setting of surgical correction of an otosclerosis‐ induced hearing loss.

### Third Window (SSCD or EVA)

The discussion regarding the necessity of preoperative imaging to exclude latent concomitant SSCD prior to stapes surgery for otosclerosis was initiated in 2010, following the case report by Hope and Fagan.^19^ They described a 30‐year‐old male with bilateral otosclerosis and an incidental unilateral left SSCD. Although his stapes surgery was uneventful and achieved near‐complete closure of the air‐bone gap, the SSCD subsequently became symptomatic, ultimately requiring resurfacing via a middle fossa approach which resolved his symptoms. The hypothesis behind this phenomenon is that after stapes surgery, the system is changed to a 3‐mobile‐window status, resulting in failure to close the air‐bone gap and leading to SSCD syndrome.^20^, ^21^, ^22^ A radiological diagnosis is recommended since SSCD with concomitant otosclerosis is usually occult due to the preoperative absence of a third mobile window.

The prevalence of otosclerosis and SSCD in the same otic capsule is not negligible. Ungar et al. recently published a meta‐analysis of over 10,000 ears. Those authors found that the incidence of SSCD in the general population compared to otosclerosis patients was similar, estimated at approximately 4%,^1^ which is in line with our current findings. The debate about the impact of occult SSCD on postoperative air‐bone gap closure and the presence of vertigo is ongoing and beyond the scope of this study. However, there is no disagreement that the informed consent discussion should include an emphasis on the increased risk of postoperative SSCD symptomatology, including vertigo and air‐bone gap and autophony.

Unfortunately, since the presence of a third window cannot be verified intraoperatively during stapes surgery, the precise NNS needed to diagnose it cannot be accurately calculated due to uncertainty about the actual sensitivity and specificity of the CT‐scan. For example, near dehiscence, which is defined as a tegmen thickness of less than 0.1 mm over the superior semicircular canal can be spuriously diagnosed as SSCD.^23^, ^24^, ^25^ Furthermore, the prevalence of dehiscent‐appearing SSC on thin‐section TB scanning is 4% to 6%, a value that is much higher than anticipated by pathologic studies.^26^, ^27^

The underlying mechanism responsible for the conductive component of hearing loss in EVA remains unclear, and several hypotheses have been proposed. Merchant and Rosowski^22^ suggested that the “third window phenomenon” within the inner ear may account for the low‐frequency air–bone gap observed in EVA. In their model, the EVA functions as an additional compliant pathway, altering inner ear mechanics such that a portion of the acoustic energy transmitted through stapes motion is diverted away from the cochlea into the vestibule. This altered energy distribution not only contributes to the air‐bone gap but may also enhance bone conduction thresholds. The incidence of concomitant EVA and otosclerosis is somewhat less defined. EVA can present in adulthood and mimic other more common pathologies, such as otosclerosis, and as a result, operated patients will probably experience no hearing gain following stapes surgery.^28^, ^29^ Inevitably, in this group of patients HR‐TBCT will be performed postoperatively as part of the inquiry as to the cause of surgical failure. Thus, the attempt to spare these patients from preoperative imaging resulted in poor surgical outcome and a postoperative imaging.

### Overhanging Facial Nerve

The low position of the FN, also referred to as overhanging FN, may cause narrowing of the oval window niche, making surgical treatment challenging.^30^ The reported incidence of FN overhang (defined as obscuring 50% or more of the oval window niche) is between 1.9% to 2.5%,^31^, ^32^ which is in line with our current findings. This anatomical abnormality can be detected preoperatively by various methods of HR‐TBCT.^33^, ^34^ Whenever present, this dehiscence leads to decreased oval window size and promontory‐facial angle. In addition, it was associated with technical difficulties, probably as a result of a limited access to the footplate and an increased risk of nerve damage.^35^ In spite of retraction of the FN and geometrical modification of the prosthesis having been described as having good postoperative motor and audiologic results, most surveyed surgeons will abort the surgery in these cases.^36^ Importantly, most of the surgeons who answered the questionnaire performed only 6 to 10 stapes surgeries annually, which is lower than the average of 12 cases annually considered acceptable for maintaining good overall surgical results (an air bone gap closure to 10 db or less in 90% of cases).^36^ It is generally accepted, however, that preoperative diagnosis of an overhang FN should change either the surgical candidacy, surgical plan, or informed consent, further supporting the performance of preoperative HR‐TBCT.

### PSA

Three main strategies have been described for the surgical management of a PSA: stapes surgery without transection of the PSA, transection of the PSA without stapes surgery, and stapes surgery combined with transection of the PSA. The association between PSA and stapes surgery is dual: PSA passage through the obturator foramen, across the stapedial footplate which then interferes with normal stapedial vibration, resulting in a conductive hearing loss w/wo tinnitus, mimicking otosclerosis. Additionally, even if there is coexisting otosclerosis, PSA may result in intraoperative dilemmas. For example. transecting a PSA could lead to profuse intraoperative bleeding^37^, ^38^, ^39^ and, in theory, cause infarction of the facial nerve or parts of the temporal lobe of the brain or the brainstem.^40^, ^41^ In spite of these considerations, some surgeons consider that stapes surgery should not be aborted in the case of an intraoperative diagnosis of PSA. However, it has never been reported whether these surgeons would operate on patients with a preoperative diagnosis of PSA. Some anecdotal reports of preoperative embolization of PSA followed by successful stapes surgery have been reported.^42^ The embryological and anatomical course of the PSA is beyond the scope of this analysis, but PSA is easily diagnosed by CT which reveals the combination of a soft tissue density mass over the stapedial footplate (100% of cases), ipsilateral absence of the foramen spinosum (100% of cases) w/wo ipsilateral aberrant carotid artery.^43^, ^44^

### Obliterative Otosclerosis

Round window obliteration is classically considered a possible cause of surgical failure.^45^, ^46^, ^47^, ^48^, ^49^ The incidence of round window obliteration is generally around 1%,^50^, ^51^, ^52^ which is in line with our findings. Round window obliteration is currently not considered a contraindication for primary stapes surgery. However, revision surgery is not recommended in the case of a poor surgical outcome, nor is round window niche drilling (associated with a 50% risk of dead ear).^53^ There are various methods to detect round window obliteration preoperatively by means of HR‐TBCT as described extensively in the literature.^49^, ^50^, ^51^, ^52^, ^53^, ^54^, ^55^ This preoperative finding should encourage the clinician to modify the informed consent regarding the overall audiological improvement success rate, but it should not modify the surgical approach.

### Far Advanced Otosclerosis

Ever since House and Sheehy utilized the terminology of “far advanced otosclerosis” in 1960,^45^ the best treatment for far advanced otosclerosis has been controversial. Although intuitively, the severe‐to‐profound sensorineural hearing loss associated with far advanced otosclerosis should be managed with cochlear implantation, the approach was initially met with skepticism and considered a contraindication to implant candidacy.^56^, ^57^ There were several reasons for the rejection: one was incomplete electrode insertion due to cochlear lumen obliteration by the otosclerotic disease, and another was altered current distribution of remodeled bone leading to higher rates of non‐auditory stimulation, particularly of the FN, even in the absence of fallopian canal dehiscence or near dehiscence.^58^, ^59^, ^60^, ^61^ Over the years, despite these reservations, a growing body of evidence has supported the notion that cochlear implantation is safe and beneficial in far‐advanced otosclerosis.^62^, ^63^ While some surgeons believed that stapedectomy is beneficial in cases of profound hearing loss due to far‐advanced otosclerosis,^64^, ^65^ others preferred cochlear implantation.^66^, ^67^ Today, it is generally accepted that stapes surgery should be the treatment of choice in ears with measurable air conduction.^64^ This controversy resulted in 2 meta‐analyses comparing audiometric results and complication rates of stapedotomy versus cochlear implantation^68^, ^69^ concluding that both stapes surgery and cochlear implantation are reliable treatment options for far‐advanced otosclerosis with similar success rates.

### Lateral Ossicular Chain Fixation

The clinical suspicion of otosclerosis is usually established by the combination of a contributory patient history combined with a conductive hearing loss and an intact tympanic membrane. Even though stapedial reflex testing is not uniformly investigated as part of the clinical diagnosis process,^15^ many studies strongly imply that acoustic reflex testing should be part of standard preoperative diagnostic workup, especially because it can discriminate otosclerosis from lateral ossicular chain fixation.^70^ Traditionally, it was believed that absence or presence of the stapedial reflex depends upon the location of ossicular chain fixation relative to the stapedius muscle insertion. Today, it is generally accepted that there is no clear‐cut dependency, and some publications reported that a stapedial reflex loss can occur in other ossicular chain abnormalities, such as malleus fixation (ankylosis).^71^ We propose that HR‐TBCT can contribute meaningfully to the differential clinical diagnosis, surgical planning and informed consent process.

### Future Directions

CBCT and photon‐counting CT are emerging modalities offering higher resolution at lower radiation doses.^72^, ^73^, ^74^, ^75^, ^76^, ^77^ While promising for temporal bone imaging, their role in surgical decision‐making for otosclerosis remains to be defined.

### Study Limitations

The main study limitation is its retrospective nature. In addition, the interpretation of HR‐TBCT may vary across radiologists and institutions, which could affect reproducibility. In addition, the study did not evaluate long‐term surgical or hearing outcomes, thereby restricting conclusions about the validity of the preoperative decision‐making process.

## Conclusion

HR‐TBCT has a useful role in the management of stapes surgery candidates. We determined, for what we believe to be the first time, that the overall NNS (95% CI) is 38 (77‐22). This imaging methodology can successfully detect complicated cases, modify informed consent, and question the benefit of surgery for high‐risk patients.

## Author Contributions

**Omer J. Ungar**, study design, conceptualization, statistical analyses, manuscript writing; **Adi Brenner**, critical revision, data collection; **Daniel Zikk**, critical revision, data collection; **Rani Abu‐Eta**, critical revision, data collection; **Yahav Oron**, critical revision, data collection; **Oren Cavel**, critical revision, data collection; **Ophir Handzel**, critical revision, data collection; **Nidal Muhanna**, critical revision, data collection; **Shiri L. Maymon**, data collection, manuscript writing.

## Disclosures

### Competing interests

None.

### Funding source

None.
